# Using the Diffusion of Innovation Theory to Understand COVID-19 Booster Hesitancy in Adults

**DOI:** 10.3390/ijerph23030327

**Published:** 2026-03-06

**Authors:** Caseem C. Luck, Sarah Bauerle Bass, Katie Joan Singley, Ariel Hoadley, Kirsten Paulus, Imani Askew-Shabazz, Whitney Cabey, Malak Abuhillo, Patrick J. A. Kelly, Maria Rincon, Heather Gardiner

**Affiliations:** 1 Department of Social and Behavioral Sciences, Temple University College of Public Health, Philadelphia, PA 19122, USA; caseemluck@usf.edu (C.C.L.); katie.singley@temple.edu (K.J.S.); kirsten.paulus@temple.edu (K.P.); iwshabazz@temple.edu (I.A.-S.); malak.abuhillo@gmail.com (M.A.); 2 Johns Hopkins Comprehensive Opioid Research Program, John Hopkins School of Medicine, Baltimore, MD 21224, USA; ahoadle3@jh.edu; 3 Temple Health Systems, Lewis Katz School of Medicine at Temple University, Philadelphia, PA 19140, USA; whitney.cabey@temple.edu; 4 Department of Behavioral and Social Health Sciences, Brown University School of Public Health, Providence, RI 02903, USA; patrick_kelly@brown.edu; 5 National Cancer Institute, Division of Cancer and Control of Population Sciences, Rockville, MD 20892, USA; maria.rincon@nih.gov; 6 Jacobs School of Medicine and Biomedical Sciences, University of Buffalo, Buffalo, NY 14203, USA; hmm6@buffalo.edu

**Keywords:** COVID-19, vaccine hesitancy, vaccine literacy, diffusion of innovation

## Abstract

**Highlights:**

**Public health relevance—How does this work relate to a public health issue?**

- COVID-19 continues to be a significant public health issue, related to both morbidity and mortality because of waning immunity from original vaccinations.
- Most people in the United States have failed to get a COVID-19 booster vaccine because of pervasive booster vaccine hesitancy, compromising public herd immunity.

**Public health significance—Why is this work of significance to public health?**

- Use of diffusion of innovation theory and qualitative methods provides key insights into how different adopter groups conceptualize barriers to COVID-19 booster vaccine uptake.
- Findings can be used to develop public health interventions that address specific concerns, especially in those least likely to get a COVID-19 vaccine booster.

**Public health implications—What are the key implications or messages for practitioners, policy makers and/or researchers in public health?**

- Given diffusion of innovation theory’s application to other health behaviors, it provides a viable framework to analyze willingness to receive COVID-19 booster vaccinations.
- Messaging about COVID-19 booster vaccinations may have to be tailored to unique concerns by diffusion of innovation adopter groups.

**Abstract:**

COVID-19 vaccine hesitancy is well documented, but less is known about booster hesitancy among fully vaccinated adults. A qualitative approach was employed to identify factors affecting COVID-19 booster hesitancy using diffusion of innovation (DoI) theory. The study was conducted in Philadelphia, Pennsylvania. In-depth interviews (*n* = 30) were done with adults, including those who had (*n* = 9) and had not (*n* = 21) been boosted. Participants were categorized into DoI adopter groups or a “refuser” group for those with no intention of getting boosted. Transcripts were analyzed using an iterative coding process with consensus and triangulation to develop thematic categories. Participants had a mean age of 41 and were 63.3% Black; 20% were classified as innovators, 6.7% early adopters, 3.3% early majority, 6.7% late majority, 43.3% laggards and 20% refusers. Three themes varied across groups: level of perceived risk susceptibility of getting COVID-19 in the future, information needs and levels of vaccine literacy, and effects of ongoing institutional mistrust. Those in the laggard and refuser groups generally had lower vaccine literacy, higher levels of institutional mistrust, and were more likely to listen to friends and family for booster advice, all consistent with DoI adopter characteristics. These differences indicate important intervention targets to promote booster uptake, especially in those who have been previously vaccinated.

## 1. Introduction

Since January 2022, the U.S. Centers for Disease Control and Prevention (CDC) has recommended an mRNA additional booster vaccine following the primary COVID-19 vaccination series [1]. Currently, CDC recommends people 12 years and older who have previously received one or more original monovalent or bivalent mRNA vaccine doses should receive one dose of any updated COVID-19 vaccine [2]. While the uptake of primary COVID-19 vaccinations has curbed both morbidity and mortality among the vaccinated [3], the emergence of variants and low booster uptake threaten population immunity [4]. For example, the Omicron variant resulted in then record-high COVID-19 case counts across immunization status in winter 2021 through early 2022. More recent SARS-CoV2 subvariants currently cause the majority of COVID-19 cases [5], most of which the initial vaccine does not adequately protect against. The only way to achieve widespread immunity from these newer variants is updated booster vaccination. But booster doses reported in fully vaccinated people remain low. As of May 2024, the number of U.S. boosted adults over the age of 18 is only 22.5%, with 41% reporting that have no intention of receiving an updated vaccine [5]. In Philadelphia, where this study occurred, only 18.2% of adults have received an updated COVID-19 booster vaccine [6].

Recent attention has been paid to COVID-19 vaccine hesitancy given concerns about waning immunity [7] from initial vaccinations. But little is known about adult booster vaccine hesitancy, especially among fully vaccinated adults. It remains unclear whether barriers and beliefs about COVID-19 booster doses vary from those associated with the primary vaccination series. The limited work that has been done regarding COVID-19 booster acceptance has been among non-US adult populations using web-based surveys [8,9,10,11,12,13], which may have limited generalizability to US adults and those facing technology barriers. One cross-sectional survey of U.S adults found that just over 20% of fully vaccinated US adults were unsure or hesitant to get a recommended booster dose [14]. Similar U.S based studies have suggested specific concerns about COVID-19 boosters, including anticipation of annual booster frequency [15], negative experiences with side effects from prior doses, uncertainties about booster side effects, chronic health issues, allergic reactions, and no initial mention of needing boosters during first vaccination [16]. Importantly, little is known about what psychosocial or “psychographic” factors related to vaccine hesitancy may be driving uptake, including perceptions, emotions, trust and experienced racism or xenophobia [7,17,18]. To that end, identifying and characterizing the attitudes and beliefs of adults vaccinated and eligible for a COVID-19 booster dose is needed to ensure the development of evidence-based communication strategies.

To address this gap, this study qualitatively explored attitudes and beliefs associated with COVID-19 booster hesitancy among fully vaccinated adults living in Philadelphia. To understand differences in perceptions, Everett Rogers’ diffusion of innovation (DoI) theory was applied [19,20]. DoI has been extensively used in public health as a framework for understanding innovation uptake. DoI provides a snapshot of groups of adopters based on different characteristics and highlights which factors influence uptake of an intervention, behavior, or innovation [21]. We used the five adopter categories—innovators, early adopters, early majority, late majority, and laggards—to specifically understand booster uptake [19,20] and added a “refuser” category for those who had no intention of being boosted. These adopter groups have certain general characteristics that can be helpful in understanding COVID-19 booster hesitancy. For example, early adopters tend to be integrated into the local social system and are opinion leaders. Those in the early majority adopt new ideas before the average member of a social system and interact frequently with peers, while the late majority are more skeptical and adopt new ideas later, often because of increasing social pressure. Finally, laggards are usually the last to adopt innovation and are likely to be suspicious of not only innovations but of innovators and change agents [19,20]. While DoI has had limited application with COVID-19 vaccine-related health interventions, prior studies [22,23,24,25] have cited the utility of DoI in conceptualizing factors associated with vaccination adoption patterns. Thus, we applied DoI to frame the understanding of potential differences in motivations for getting boosted between those who had and had not been boosted that could inform a broader survey of vaccinated adults.

## 2. Materials and Methods

Philadelphia residents (N = 30) participated in a one-on-one semi-structured interview from May through August 2022. A cross-sectional sample of boosted (*n* = 9) and un-boosted (*n* = 21) participants were recruited, with the goal to understand drivers of intent to receive a booster dose, and elucidate psychosocial, experiential, and structural barriers to receipt of a COVID-19 booster vaccine. Participants were recruited through social media and newspaper print ads and eligibility was verified over phone and by a valid Philadelphia mailing address. Prospective participants were eligible if they were at least 18 years of age, were a resident of Philadelphia, and reported being fully vaccinated for COVID-19 (i.e., one dose of Johnson and Johnson or two doses of Pfizer or Moderna vaccine). Eligible participants were sent an informed consent form via email/mail and, once consented, scheduled an interview date and time convenient to them. Interviews were offered both virtually on Zoom and in person. Participants also completed a short demographic survey. Individual interviews were conducted by study staff who have extensive experience with qualitative research methods [26,27,28] and took from 30 min to 1 h depending on responses. Interviews occurred until saturation was reached in the boosted and un-boosted groups, and when similar decisional factors concerning both initial vaccine and booster decisions within each DoI adopter group were found. Though some groups had small membership (i.e., 2), it was felt that decisional factors were aligned because these groups were those who had been boosted and there were not significant differences across these early adopter categories. Thus, it was deemed not necessary to recruit more participants in these categories. All participants were compensated with a gift card as an incentive for participation. The Temple University Institutional Review Board approved the study (Protocol #29430).

### 2.1. Interview Guide

The interview guide was crafted to prompt participants to discuss their perceptions of the COVID-19 boosters, including how COVID-19 impacted their lives, where they went to receive trusted health information, and various factors that drove the decision to get a booster or not (see Table 1 for interview questions by domain). Questions were broad to allow participants to discuss their own perceptions of the booster and to illicit potential barriers to uptake.

### 2.2. Analysis

Interviews were audio recorded, transcribed and analyzed using an iterative coding process with consensus and triangulation to develop thematic categories using Dedoose (9.0.107). DoI served as the theoretical basis for the analysis. Each participant was assigned to a specific adopter group based on the timeframe of their initial COVID-19 vaccine and booster vaccine decision. Specifically, early adopters were those that got a booster as soon as it was available; late majority were those who had not received the booster yet but indicated they were still open to getting it. An additional “booster refuser” group was added to capture participants who noted they had no intention of getting a booster dose, to differentiate them from the “laggard” group that did not have any intentions on receiving a booster vaccine but would under certain circumstances. Other DoI constructs, such as attributes of the innovation or social system, were not used in analysis. A codebook was developed through an initial open coding process that informed the development of a standard codebook, which was then applied across all DoI groups by four trained coders. The analysis was guided by Braun and Clarke [29]’s suggested stages: familiarization with data, generation of codes, thematic search, and meaning derivation from the coded data across the DoI adopter groups. Any discrepancies were decided through discussion, including identifying the appropriate adopter group for participants. Thereafter, a comparative analysis between the DoI groups’ unique decisional factors associated with their booster vaccine decision determined common thematic insights. The coding process was documented and reviewed by study staff to maximize the reliability and validity of the analysis. Each group was assessed for consistency using DoI characteristics and their stated decisions about both the initial vaccine and boosters to determine if saturation had been achieved.

## 3. Results

### 3.1. Demographics and Interview Themes

Our study sample ranged in ages from 18 to 72, with a mean age of 41 (SD: 15.39). More than half of the participants identified as Black/African American (*n* = 19, 63.3%) and 46.7% had a college degree or higher. In terms of their COVID-19 booster vaccination decision, 20% were innovators (*n* = 6), 6.7% were early adopters (*n* = 2), 3.3% were early majority (*n* = 1), 6.7% were late majority (*n* = 2), 43.3% were laggards (*n* = 13), and 20% were booster vaccination refusers (*n* = 6). Table 2 presents the demographic characteristics of the study sample. Three common themes were identified, with each DOI group communicating varying levels of perceived risk susceptibility, information needs about booster side effects/vaccine effectiveness, and skepticism and mistrust towards institutions such as the government and the U.S healthcare system. Sample quotes by adopter groups are shown in Table 3.

#### 3.1.1. Various Levels of Perceived Risk Susceptibility

Participants from the earlier adopter groups in the sample (booster innovators, booster early adopters, and booster early majority) expressed little to no differences in their reasons for being boosted and had few concerns about booster vaccines. However, they did note that knowing about the side effects and vaccine ingredients was important to them prior to getting their booster shot. Relatedly, participants’ perceived risk susceptibility of contracting COVID-19 was higher compared with later adopter groups. Many mentioned that “protecting family” was a central factor in both their primary and booster vaccination decisions. One participant explained: “There’s no cure for this. So, like, if there’s something with the potential to, you know, save my life, or my children’s lives, or, you know, make it not that sick, then let’s probably do that. That was literally my decision-making process.” (Booster Innovator, Interview 11).

Many of those in the earlier adopter groups had some direct connection to the healthcare system. Some worked or had family and friends who worked in the healthcare system, leading the group to have high vaccine knowledge. For example, innovators often conceptualized the primary vaccine and additional boosters as a probable continuous occurrence moving forward rather than a single vaccine event. Similar comments were expressed in the other two early adopter groups.

Participants in the booster late majority group had not yet been boosted but voiced intention to do so soon. When discussing risk susceptibility, participants were aware of their moderate to high risk of contracting COVID-19 because of their work/school environments. Participants shared similar concerns about how not being vaccinated and/or boosted would affect their ability to secure employment and participate in on-campus university student life.

None of the participants in the booster laggard group had received an additional booster vaccine at the time of the interview and most were less enthusiastic about getting it in the future. Interestingly, 8 of the 13 of these participants were categorized as vaccine innovators, early adopters, and early majority adopters for their initial COVID-19 vaccination decision. However, when it came to their booster decision, concerns regarding potential side effects, stemming from negative experiences with their initial vaccination series, were evident. One participant said, “I did not like that second time. That’s what’s holding me back from taking a booster because I don’t want to. If I get the booster, am I gonna get sicker than I did the second time?” (Interview 19).

For the booster refuser group, low perceived susceptibility of getting sick from COVID-19 because of prior exposure, information fatigue, and ongoing treatment for long-standing health conditions were reasons noted for not being boosted. One participant commented, “I feel like I’m vaccinated. I’m not really that worried about it.” (Interview 25). While some members of this group communicated that they would not receive the booster under any circumstance, others mentioned that they would only receive additional booster vaccine dosages if they contracted COVID-19. One participant described that they would only receive an additional booster vaccine if it provided lifelong protection. They said: “If they had a booster that was effective, and perhaps even permanent, something that will protect you from all of the, or at least most of, the variants that are emerging, I would probably be more inclined to getting the booster.” (Interview 27).

#### 3.1.2. Information Needs About Booster Side Effects/Vaccine Effectiveness

When discussing where participants sought information about COVID-19 boosters, earlier adopter group participants tended to search for information about booster vaccinations from three sources: peers, government health agencies (i.e., CDC), and their healthcare provider. These information sources were perceived to provide credible information about booster vaccines, whereas social media and certain news channels were deemed not credible information sources to learn about initial vaccine and additional booster doses.

The two participants in the late majority group were also the youngest (18 and 19 years old), both having recently completed their high school education during the height of the COVID-19 pandemic and, at the time of their interviews, were either entering the workforce or attending university. In turn, parental approval or disapproval of the COVID-19 vaccine was a key determinant for intended booster uptake. One participant described how their mother’s lack of support and conspiratorial beliefs about the booster vaccine made them hesitant about their decision to ultimately get boosted. They said: “My mother is not as enthusiastic… It’s harder for me to just go up and do that knowing it’s going to make her upset… she’s so against it and has made that so clear. So that’s why, it also is what played into my like hesitancy.” (Interview 7).

In comparison, the other participant explained how the encouragement from their family members helped shape their positive attitudes about the vaccine. They said, “I was thinking about getting it because my family was all vaccinated… They just encouraged me, they said it doesn’t hurt. Nothing major will happen.” (Interview 10).

Concerns about booster side effects and needing more information were relative to participants’ initial experiences with their primary vaccination series and experiences with contracting COVID-19. Generally, those participants who were more likely to want to get the booster were able to rationalize vaccine side effects because of the minimal effects they experienced from the initial vaccine, or because of their awareness of how vaccines build immunity. Those who experienced side effects were more likely to either need more information or be less inclined to get the booster.

In the booster laggard group, many believed they needed more information about booster vaccines before they would go get one. Some participants leaned heavily on their primary care physicians as trusted sources of information, including their endorsement for the recommended booster dosages. “I have to talk through with my physician. I think it may be beneficial for me to get it at some point, I may consider getting it, but I’m not quite there yet.” (Interview 17).

Other participants in the booster laggard group were skeptical about the utility of the additional booster dosages compared with the other adopter groups. One participant commented, “Yeah, because I don’t understand what’s the booster for?” (Interview 19). Often, participants’ rationale for their concerns were rooted in lower vaccine-related health literacy.

Participants tended to rely on anecdotal information from their peers and family members, whom they perceived as credible information sources to shape their opinions of the booster. However, some participants commented about how these testimonials prompted them to make the decision to receive a booster shot, especially if their peers or family members experienced minimal to no side effects: “Yeah, usually through my wife who’s a nurse, that’s a very reliable information…Yeah, I think I said my wife was my only, you know, source of information all through till now so I would just, you know, ask her if I had any doubts… What matters was it [information about COVID-19 vaccines] was coming from her…” (Interview 12).

When discussing credible information sources, the booster refuser group participants said they often relied on internet search engines (Google), testimonials from peers, and TV news media to gather information about the booster vaccines. One participant commented they were not sure where to look for reliable information about the booster vaccine. They said, “I seen some things, from what Google provided and from what people were posting on social media. But I didn’t really get a clear answer, and I didn’t know where to look.” (Interview 27).

#### 3.1.3. Institutional Skepticism and Mistrust

In the earlier adopter groups, discussions of skepticism of the U.S. government and not really knowing what it was doing regarding COVID-19, were prevalent. However, trust in the healthcare system was common. In comparison, perceptions of booster vaccines in the booster refuser group led to tangential discussions of concerns about their trust in the United States healthcare system, including mistrust of institutions as a whole and beliefs in conspiracies. One participant highlighted their concerns about vaccines in general, stemming from a long-standing distrust of the healthcare system because of historical incidents of medical malice toward the African American community and individual negative encounters. They said, “A lot of that research is, for me, is skewed. I don’t find it to be extremely reliable enough. And I know that in the past, especially when it comes to African American people, we were thought of as having no pain. And even when you look at certain medical guidelines, it’s, it’s not based off an African American body type.” (Interview 8).

Another participant asserted their belief that the COVID-19 pandemic was fabricated by the U.S government. They said, “this whole thing is made up. I don’t think it’s real, you know.” (Interview 20). The participant went on to comment that they believed the pandemic was a ploy by the U.S government to “control and scare people,” and, if given the opportunity, they would attempt to dissuade people from receiving booster vaccines. They said, “just don’t get no vaccines or boosters at all, because it’s a whole conspiracy. And if your health is already fine, then just, you know, stick with that good health.” (Interview 20). These participants expressed high levels of mistrust about COVID-19 and the vaccines.

## 4. Discussion

Results from our study provide insights into key drivers of COVID-19 booster hesitancy, including the importance of specific psychographic variables, such as risk susceptibility and institutional mistrust. While prior studies have explored various demographic factors (e.g., gender, age, health status) in relation to initial COVID-19 vaccination intention [30,31,32], such results are limited in explaining why a portion of people are still hesitant or do not intend to receive additional recommended vaccinations [30,33], especially if they are already vaccinated. Findings provide key insights into salient factors that motivate or hinder uptake of booster doses that could be addressed in health education campaigns to promote uptake of COVID-19 vaccination. Secondly, results helped elucidate how these drivers impacted decisional outcomes in participants in different diffusion of innovation adopter groups, something not previously seen in other studies.

When looking across the DoI adopter groups, information appraisal was a consistent variable in participants’ risk assessment concerning booster vaccination. Generally, the “need for more information” about booster vaccines was prompted by either conflicting information from various media news sources about vaccine development or anecdotal information from peers about side effects/booster vaccine effectiveness. Primary care providers were often identified as a credible and trusted source for information about additional booster vaccines, especially in earlier adopter groups. Similar findings have been reported in a recent study [34] that suggests physician recommendations for COVID-19 vaccination may reduce vaccine hesitancy among individuals who are unsure about getting vaccinated and reduce the spread of misinformation about vaccination. However, it was also clear that those in the booster refuser group were more likely to be distrustful of doctors or healthcare institutions, making medical mistrust an important variable to consider in understanding and addressing COVID-19 booster hesitancy.

One interesting finding was that participants’ individual decisional factors for getting or not getting COVID-19 booster vaccinations were often independent of the factors for receiving their initial vaccination series, regardless of their DoI group. Our data suggests that participants perceived the initial COVID-19 vaccine series and the additional booster vaccines as entirely separate and many developed new concerns about the booster they had not previously had about the primary vaccinations. This was especially true in the laggard group, where the majority were categorized as innovators, early adopters or early majority in their initial vaccination decision. This suggests that initial communications about the COVID-19 vaccines insufficiently prepared people for the possibility of annual COVID-19 immunizations or that there was an “adoption fatigue” that occurred, affecting how people were thinking about the booster vaccinations. Although less is known about the relationship between vaccine knowledge and vaccine intention concerning COVID-19 boosters, prior studies about HPV vaccination intention have shown that high vaccine-related health literacy is a key indicator for vaccine uptake [35,36]. Other studies [33,37] have noted that when people have higher health literacy about vaccines, they are more likely to see minimal risk in receiving a vaccination compared with people with lower vaccine health literacy [38,39,40]. Thus, while people may have felt comfortable getting the initial vaccine, the relative lack of attention in public discourse about the booster may have decreased booster health literacy, especially in the laggard and refuser DoI groups, impacting acceptance.

Institutional distrust and beliefs in conspiracies were important in shaping some participants’ perceptions of risk and the utility of additional booster vaccines. While not widespread, some booster refuser participants noted their beliefs that both the initial and additional COVID-19 vaccines were not effective in protecting against getting sick. Rodgers and Shoemaker [41] suggest that later DoI adopter groups generally have higher levels of skepticism about behavior adaptations. Relatedly, scholars [42] have identified that one of several causes for conspiratorial thinking is situational uncertainty, for example the potential side effects caused by the COVID-19 booster vaccine. Given the extensive amount of misinformation/disinformation that has circulated in the past four years concerning the origins of COVID-19 and the development of the vaccines [43,44], it is clear this confirms distrust in some of those in these later adopter groups.

Finally, results from our study provide insights into viable pathways to disseminate information about COVID-19 boosters. U.S-based communication campaigns [45,46] have generally focused on addressing structural barriers associated with vaccine uptake in specific racial/ethnic groups. Our findings suggest that such barriers were less prevalent compared with participants’ internalized perceptions associated with the booster vaccine. Prior vaccination interventions have utilized various social media outlets and community-based organizations to disseminate educational information about booster vaccines [47]; similar suggestions were shared by participants in our sample, regardless of DoI adopter category. Participants noted that information on social media could be an important way to share information and should use clear content language, use credible social media accounts, and have endorsements from reputable community members/medical practitioners. This would be important to increase credibility of this information, since many also noted social media as generally not a credible source of information.

Overall, these results suggest a more informative approach for intervention and public health practice would be to identify and address more psychographic drivers and barriers to booster vaccination intention, especially in later adopter groups [30]. Applying DoI theory to conceptualize participants’ primary drivers for their booster vaccination decision allowed for segmenting participants into adopter groups by their initial and booster vaccination decisions and comparing perceived barriers and facilitators to uptake, a novel approach. While this sample was urban and predominately Black, it may be that findings would not translate to other geographically diverse regions, such as rural areas. However, using DoI adopter categories actually allows for characterizing population segments across geographies in a more standardized way. Previous research indicates that innovations are adopted in urban/metropolitan areas before spreading to rural areas [48]. But since the majority of the sample was actually categorized as laggards in this urban population, we believe that using DoI provides a viable and important framework [22,23,49] to analyze willingness to receive additional COVID-19 vaccinations [24].

There are limitations to this study. Findings may not reflect specific concerns seen in rural areas or outside of the Northeastern United States. Furthermore, over 63% of our sample identified as African American, who often are more likely to be concerned about discrimination and have higher levels of medical or institutional mistrust [50,51]. This may skew results to emphasize mistrust as a key decisional variable for getting boosted. However, the demographics of our sample are similar to those in Philadelphia [52]. Due to the cross-sectional nature of our sample, we also had almost half of the sample characterized as laggards or refusers. This, along with the small number of participants in some adopter groups, may not adequately reflect the population as a whole. Finally, since our data were collected, additional booster vaccinations have been recommended. Hence, some findings from our study may not reflect specific concerns associated with updated COVID-19 vaccines.

## 5. Conclusions

Our research leveraged diffusion of innovation Theory to conceptualize decisional factors associated with the adoption of COVID-19 booster vaccines. By understanding how messaging needs to address concerns by adopter categories, more tailored intervention messages can be crafted to increase booster vaccine intent.

## Figures and Tables

**Table 1:** Interview guide domains.

Domain	Sample Questions
**COVID-19 Experiences**	Could you tell me a bit about what the impact of COVID-19 has been on yourself and the people in your community? ○ PROBE: Family, community, work?
**Information Sources**	Where do you get most of your health information? ○ PROBE: Healthcare providers, family/friends, government officials, news like on television or radio, the internet including social media? 2. Are these the same sources you go to for information about the COVID-19 vaccines, including boosters? ○ PROBE: *if no*: What other sources do you go to (i.e., healthcare providers, family/friends, government sources, news like television/radio, the internet including social media)? ○ PROBE: Does it make a difference to you if your doctor recommends you get a vaccine? 3. Who or what sources do you trust the most? ○ PROBE: Why? How do you know these are good sources of information? 4. Who or what sources do you trust the least? ○ PROBE: Why? How do you know these are not good sources of information?
**COVID-19 Vaccine Beliefs and Experiences**	Which vaccine did you receive (*Moderna, Pfizer, Johnson and Johnson*)? I want you to think back to when you were deciding to get vaccinated. Please walk me through your thinking. ○ PROBE: What concerns did you have about the vaccines, if any? ○ PROBE: What concerns did others around you have? Same or different? 3. What helped you decide to get vaccinated? ○ PROBE: Were you worried about your health? Were you affected by a mandate for work or other activities? Did you consider our social responsibility to others in making the decision?
**Booster Perceptions**	Tell me what you know about boosters. ○ PROBE: Why do you think it is recommended that people get booster shots? 2. What are people in your community saying about booster shots? ○ PROBE: Have people in your life been boosted (family, friends, etc.)? IF BOOSTED What was your experience like? ○ PROBE: Did you experience side effects? Did you have to take time off from work/school? ○ PROBE: Did you have any difficulties finding a booster, scheduling a booster, getting to a booster clinic, or any other issues? IF NOT BOOSTED What things are keeping you from getting a booster? ○ PROBE: Fear of side effects/recall side effects from other doses, inconvenience/lack of time? 2. What would make you interested in getting a booster? ○ PROBE: Do you have any unanswered questions about the booster or any other information that you need to help you to make your decision?
**Messaging about Vaccines/Boosters**	How do you think we should communicate information about boosters to people? What do you think about us using social media to share information about the boosters? ○ PROBE: What makes a social media post about boosters seem more credible or trustworthy? 3. What do you think about us using things like billboards, transit ads, and posts in the newspaper to get information out about the booster? ○ PROBE: Do you think there are certain communication channels that make the most sense to use for your community? 4. We are also going to be partnering with different community organizations and spokespeople. What thoughts do you have about receiving information about boosters from: ○ Medical professionals? ○ Faith-based leaders? ○ Block captains? ○ Local celebrities? ○ Other community members?

**Table 2:** Participant demographics by adopter group.

Baseline Characteristics	Innovators	Early Adopters	Early Majority	Late Majority	Laggards	Refusers	Full Sample
	*n* (%)	*n* (%)	*n* (%)	*n* (%)	*n* (%)	*n* (%)	*n* (%)
**Gender**							
Female	4 (66.7%)	1 (50.0%)	1 (100%)	1 (50.0%)	5 (15.4%)	3 (50.0%)	15 (50.0%)
Male	1 (16.7%)	1 (50.0%)	0 (0.00%)	1 (50.0%)	8 (61.5%)	3 (50.0%)	14 (46.7%)
Not Specified	1 (16.7%)	0 (0.00%)	0 (0.00%)	0 (0.00%)	0 (0.00%)	0 (0.00%)	1 (3.3%)
**Age (Years)**	31.25 (SD 8.30)	32.50 (SD 6.36)	28.00 (SD 0)	18.50 (SD 0.71)	49.23 (SD 15.23)	45.83 (SD 11.37)	41.78 (SD 15.39)
**Ethnicity**							
Hispanic/Latino	1 (0.00%)	0 (0.00%)	0 (0.00%)	0 (0.00%)	1 (7.7%)	1 (16.7%)	2 (6.7%)
**Race**							
Black/African American	3 (50.0%)	2 (100%)	1 (100.%)	1 (50.0%)	8 (61.5%)	4 (66.7%)	19 (63.3%)
White/Caucasian	2 (33.3%)	0 (0.00%)	0 (0.00%)	1 (50.0%)	5 (38.5%)	0 (0.00%)	8 (26.7%)
Multiracial/Multi-ethnic	1 (16.7%)	0 (0.00%)	0 (0.00%)	0 (0.00%)	0 (0.00%)	1 (16.7%)	2 (6.7%)
Not Specified	0 0.00%	0 (0.00%)	0 (0.00%)	0 (0.00%)	0 (0.00%)	1 (16.7%)	0 (0.00%)
**Highest Educational Level**							
Less than High School	0 (0.00%)	0 (0.00%)	0 (0.00%)	0 (0.00%)	2 (15.4%)	1 (16.7%)	3 (10.0%)
Finished High School/GED	0 (0.00%)	0 (0.00%)	0 (0.00%)	1 (50.0%)	2 (15.4%)	2 (33.3%)	5 (16.7%)
Technical or Vocational School or Community College	1 (16.7%)	0 (0.00%)	0 (0.00%)	0 (0.00%)	1 (7.7%)	1 (16.7%)	3 (10.0%)
Some College	1 (16.7%)	0 (0.00%)	0 (0.00%)	1 (50.0%)	2 (15.4%)	1 (16.7%)	5 (16.7%)
College Degree or Above	4 (66.7%)	2 (100%)	0 (100%)	0 (0.00%)	6 (46.2%)	1 (16.7%)	14 (46.7%)

**Table 3:** Sample quotes by adopter groups.

	Sample Quotes
**Booster Innovators** Description: Boosted on own volition, no questions asked. No expressed concerns or reservations and only articulated benefits of booster. Boosted when first able to/recommended.	“I am very lucky, I had no problems, no side effects [Booster vaccine] …I didn’t get sick, didn’t have any of these weird side effects. So, I’m kind of an anomaly because I know like, my mom, for example, she got like a fever, that whole thing and many, many other people got got stuff so.” (Interview 2)
	“So, like, I’d rather have some protection, and none at all. wherever that is, I want that protection [booster vaccine].” (Interview 9)
**Booster Early Adopters** Description: Sees value in boosters as the best thing to do to extend the protection of original vaccine series and articulates that it is best to follow the recommendations from leaders to help society. Discussed with others and sought out booster when available.	“I mean, I think I think all of it is kind of like, it’s gonna all be the same on board. Like it was like my parents were talking about the boosters. And I know, like, older people were getting it. And so like my grandparents, I think were the first in our family to get their booster shots. And like, we had a group chat, and they like were encouraging all of us to go. They were like, yeah, we just got our boosters.” (Interview 5)
	“Being that this was something that was spreading quickly, people were dying it like crazy rates hearing, like, you know, testimonials and videos from frontline staff and hospitals and nurses and all kinds of stuff like, oh, this is, this is crazy. And we’re, I was I was watching some show. Some, like medical show, drama, and this kid that came in with an illness that there was a vaccine for. And there was a lot of like, back and forth about like, well, there’s, there’s something that could help you and you’re not taking advantage of it. Like, why would you do that? And from, for me, that it just, it really stood out to me, like, I felt really convicted in that moment.” (Interview 11)
**Booster Early Majority** Description: Had questions asked and answered. May have spoken with doctors or other healthcare people to have questions answered. Did after others they trust (friends, family, doctor) started getting boosted.	“No, and I think that was I think there also was like that being not being a big decision. For me, it made it easier to focus on just the well-being of me mentally, and me and my child and I. Because I, I actually work with family at home health Aid. And I work in the office, so I don’t have to work around patients. I just worked in office with two of us. Okay, so was it mandated for, for me to get it.” (Interview 13)
	“My decision came from at first not getting it because I mean, that may come from getting it while pregnant. I didn’t know, the true measures, it would have all my son. And then as I gave birth, I was so skeptical just because of breastfeeding. I wasn’t really considering formula feeding him and I didn’t want anything to jeopardize that at least not for the first six months of his life. So that was that was it and then once I did research that I couldn’t really find much on it affecting, you know, breastfeeding, that was my decision to like, Okay, I feel better now.” (Interview 13)
**Booster Late Majority** Description: Out of sight out of mind. Low perceived risk of COVID or wanted to wait and see the evidence or see how many others were getting boosted. May have had concerns about speed of development, side effects, long term effects. May be moved to uptake by testimonials from friends, family. May be moved to uptake if an improved booster dose was available.	“So, like now I’m in the same position as it was last summer where I have to make the decision again, to do it without her [Mother], I guess consent or approval. So, I do want to get my booster and I’m planning on doing it soon. It’s just a matter of like, having to have that uncomfortable conversation again with her [Mother].” (Interview 7)
	“No, I just think that the booster is just um, it helps more. With that in the booster will help more than just a shot by itself.” (Interview 10)
**Booster Laggards** Description: Not boosted and don’t really plan on being boosted unless forced to for work. Usually has serious questions about the need for booster and questions its need.	“That’s why I’m skeptical about-I want to get the booster shot, and then I don’t. Because what good does it do if some people are getting the shots and they’re getting sick?” (Interview 19)
	“I don’t know. I feel like after I got the two shots, I felt like that was enough for me.” (Interview 18)
**Booster Refusers** Description: Under no circumstances will they receive a booster. Would give up employment/other loss over getting boosted.	“And it’s like now, it’s like, now it’s like, they got all these different viruses and stuff like that. It’s like, they just come up with all kinds of like, you know, bull crap, you know?” (Interview 20)
	“The only way I can think of any little bit making me the slightest bit curious is if somehow someway, years down the line, it came out that there was a study of somehow this vaccine and this booster helps the human body in some kind of way.” (Interview 8)

## Data Availability

The qualitative datasets generated and/or analyzed during the current study are not publicly available due to participant privacy and confidentiality but are available from the corresponding author on reasonable request.

## Abbreviations

The following abbreviations are used in this manuscript:

COVID-19	Coronavirus Disease of 2019
DoI	Diffusion of Innovation
